# Transcriptomic and Genomic Approaches for Unravelling *Candida albicans* Biofilm Formation and Drug Resistance—An Update

**DOI:** 10.3390/genes9110540

**Published:** 2018-11-07

**Authors:** Pei Pei Chong, Voon Kin Chin, Won Fen Wong, Priya Madhavan, Voon Chen Yong, Chung Yeng Looi

**Affiliations:** 1 School of Biosciences, Faculty of Health and Medical Sciences, Taylor’s University Malaysia, Subang Jaya, 47500 Selangor, Malaysia; VoonKin.Chin@taylors.edu.my (V.K.C.); PhelimVoonChen.Yong@taylors.edu.my (V.C.Y.); ChungYeng.Looi@taylors.edu.my (C.Y.L.); 2 Department of Medical Microbiology, Faculty of Medicine, University Malaya, 50603 Kuala Lumpur, Malaysia; 3 School of Medicine, Faculty of Health and Medical Sciences, Taylor’s University Malaysia, Subang Jaya, 47500 Selangor, Malaysia; Priya.Madhavan@taylors.edu.my

**Keywords:** *Candida albicans*, biofilm, antifungal resistance, transcriptomics

## Abstract

*Candida albicans* is an opportunistic fungal pathogen, which causes a plethora of superficial, as well as invasive, infections in humans. The ability of this fungus in switching from commensalism to active infection is attributed to its many virulence traits. Biofilm formation is a key process, which allows the fungus to adhere to and proliferate on medically implanted devices as well as host tissue and cause serious life-threatening infections. Biofilms are complex communities of filamentous and yeast cells surrounded by an extracellular matrix that confers an enhanced degree of resistance to antifungal drugs. Moreover, the extensive plasticity of the *C. albicans* genome has given this versatile fungus the added advantage of microevolution and adaptation to thrive within the unique environmental niches within the host. To combat these challenges in dealing with *C. albicans* infections, it is imperative that we target specifically the molecular pathways involved in biofilm formation as well as drug resistance. With the advent of the -omics era and whole genome sequencing platforms, novel pathways and genes involved in the pathogenesis of the fungus have been unraveled. Researchers have used a myriad of strategies including transcriptome analysis for *C. albicans* cells grown in different environments, whole genome sequencing of different strains, functional genomics approaches to identify critical regulatory genes, as well as comparative genomics analysis between *C. albicans* and its closely related, much less virulent relative, *C. dubliniensis*, in the quest to increase our understanding of the mechanisms underlying the success of *C. albicans* as a major fungal pathogen. This review attempts to summarize the most recent advancements in the field of biofilm and antifungal resistance research and offers suggestions for future directions in therapeutics development.

## 1. Introduction

*Candida albicans* is the leading etiological agent for fungemia and disseminated candidiasis, which are associated with high mortality rates. According to statistics provided by the Centre for Disease Control, *C. albicans* is the third most commonly isolated microbe from bloodstream infections among hospitalized patients in the US [1]. The success of this eukaryotic microbe in causing a myriad range of human infections from superficial skin and nail infections, oral and vaginal candidiasis, to the more serious invasive candidemia and deep organ infections, is in part due to its arsenal of virulence factors and its morphology switching capability. Unlike most other fungi, *C. albicans* is able to exist in yeast, pseudohyphal as well as hyphal forms depending on the in vivo surrounding environment or in vitro culture conditions.

This versatile fungus is able to grow in biofilms on medical devices such as intravenous catheters, urinary catheters, heart pacers and other equipment that is in contact with biological fluids or organs. A huge problem encountered by clinicians treating invasive candidiasis is the enhanced antifungal drug resistance displayed by *Candida* sp. biofilms. Indeed, *C. albicans* biofilm cells have been reported in multiple studies to display up to 1000-fold greater drug resistance than planktonic, non-biofilm cells [2,3,4]. Globally, the impact of medical device-related candidiasis is undeniably serious considering the high mortality and morbidity rates ascribed to these infections that are often recalcitrant to routine antifungal therapies.

In this review, we summarize the switch from commensalism to colonization and active infection for *C. albicans* in host cells and discuss the various stages, biochemical processes and molecular changes that are essential for biofilm development and pathogenesis. The intricate transcription regulatory networks that play a critical part in biofilm formation are discussed. Next, drug resistance associated with biofilm growth of *C. albicans* will be dissected. A section will be dedicated to the chief genomic differences observed between *C. albicans* and its relatively less virulent close relative, *C. dubliniensis*. This is in line with our efforts to understand the inherent genetic factors that contribute to the success of *C. albicans* as a human pathogen. Recent studies, which report the transcriptomic analysis of genes and metagenomic profiling of antifungal drug resistance related to biofilms, are also highlighted. The final section of this review focuses on the strategies for future research on targeted therapeutics that could combat *C. albicans* biofilm formation.

## 2. Morphology Switching and Pathogenesis of Biofilm Formation

Owing to its dimorphic switching property, *C. albicans* is able to switch from a yeast to a hyphal form thereby exiting the “harmless” commensal stage to become a pathogen. In addition, the fungus possesses the trait of biofilm development; another major contributor to its pathogenesis. Normally, in healthy hosts, *C. albicans* is a commensal microbe that inhabits mucosal surfaces especially in the intestines and is almost ubiquitous in the human microbiome. Factors such as the normal microbial flora, innate immunity and also epithelial barriers prevent *C. albicans* from overgrowing or invading the deeper layers of skin or penetrating the intestinal barrier. Constant interaction between the fungus and the host immune system is believed to take place during this commensal stage [5].

During the transition from commensalism to pathogenesis, three distinct yet dynamic stages are seen, namely (i) adhesion, (ii) invasion, and (iii) damage [6]. Wächtler and colleagues were the first to show that the three stages are mediated by distinct factors. In the adhesion stage, factors that play a crucial role include the adhesins from the Als family and the cell wall components Hwp1 and Als3 [6]. The adhesion factor Eap1 was separately shown by another study to be involved in adhesion [7]. Several of these adhesins are linked to the cell membrane whereas others are linked to the cell wall via glycosylphosphatidylinositol moieties. In a study by Wächtler, a *C. albicans* microarray from Eurogentec (Belgium) was used for transcriptional profiling of genes during the adhesion, invasion and damage stages in interactions with oral epithelial cells. Many hyphal associated genes including *ALS3*, *HWP1*, *ECE1*, *SOD5*, *PHR1*, *PRA1*, and *RBT1* were found to be upregulated when in contact with the oral epithelial cells [6].

The Hwp1 and also the Als3 proteins are produced predominantly during hypha formation, to allow for the adhesion of the fungal cells either to the host cells or to a substrate surface [8]. In the invasion stage, a different set of genes are expressed; although the common involvement of Als3, a multifunctional adhesin and invasin protein, is also present. The proteases such as secreted aspartyl proteinases (Saps) and phospholipases have long been known to be crucial players in the hyphal invasion of host cells. On the other hand, the onset of host cell damage is a key feature of pathogenesis. Tissue damage occurs when *C. albicans* hyphae penetrate deep into or through the epithelial layer (interepithelial layer), a process facilitated by secreted hydrolases [9]. A previous study by Wächtler [6] suggested that Icl1, Sod5 and Yhb1 are involved in the active infection of epithelial cells in particular during epithelial damage. In a recent study, Allert and coworkers screened libraries of *C. albicans* deletion mutants in a quest to delineate genes involved in host epithelial damage and translocation through the intestine barrier. They found that candidalysin, a peptide toxin of *C. albicans*, is crucial for this process [10].

Biofilms are complex three-dimensional structures that are composed of a core microbial cell community (either a single species or a mixed species) attached to host tissue or abiotic surfaces surrounded by an extracellular matrix (ECM) of polysaccharides that provide a shield or scaffold for the microbes beneath [11,12]. Not surprisingly, a biofilm provides protection against antimicrobials for the microbes associated with it, and thus biofilm-associated infections are notoriously difficult to treat. Among the clinical isolates of *Candida* species and even across different species, there is great heterogeneity in terms of the biofilm forming capability. The ability of clinical *Candida* isolates to form a biofilm has been shown by many studies to correlate with a higher virulence and hence an increased mortality [13,14,15].

In *C. albicans* biofilm formation, several stages are distinguishable: (i) adhesion, (ii) initiation, (iii) maturation, (iv) dispersal; which typically progress in sequence over a period of 24–48 h [16,17]. In the adhesion step, single *C. albicans* yeast cells adhere to the substrate to form a basal layer of yeast cells. The cell proliferation phase ensues followed by filamentation whereby the yeast cells begin to elongate and develop into filamentous hyphae. This is the initiation step whereby the cells change their morphology and invade either the host mucosal site or plastic or other polymer surfaces in inert medical devices. An arsenal of hydrolytic enzymes such as proteinases, haemolysins, and phospholipase are secreted by *C. albicans*, which enables the fungus to invade host tissue or other solid substrates. The secreted aspartyl proteinases (Saps), comprising a family of ten genes (*SAP1–10*), are the most well studied among the many secreted enzymes [16,17].

In the maturation step, the production of hyphae is a key feature accompanied by the secretion of ECM of polysaccharides. The biofilm ECM of *C. albicans* is complex, where the major polysaccharides include α-mannan, β-1,6 glucan and β-1,3 glucan [18]. Among these, although β-1,3 glucan is a minor constituent, it is the chief matrix polysaccharide linked to biofilm resistance to antifungals as it could block drug diffusion [19]. The hyphal invasion into tissues is driven by physical hydrostatic forces (turgor), which drive the cytoplasmic forces. The cells could communicate with other cells through quorum sensing mechanisms and one of the most studied quorum sensing molecules that could regulate biofilm formation is farnesol [20].

Finally, in the dispersal stage, lateral yeast cells are released from the matured biofilm and are then able to disseminate to distant sites to initiate a new cycle of biofilm formation. The dispersal stage of biofilm is of immense clinical relevance, as the newly released cells from the mature biofilm located in either indwelling catheter or an infectious nidus are able to not only initiate new rounds of biofilm formation but also enter into the bloodstream to establish a distant focus of infection. This is the reason why biofilm formation is closely associated with candidemia and disseminated invasive candidiasis clinically [21]. Importantly, a previous study had shown that the dispersed cells are predominantly yeast cells, with associated enhanced adherence, filamentation capacity, biofilm formation, increased resistance to azole drugs, and are more pathogenic than their planktonic counterparts [22]. Interestingly, in the latest study by Uppuluri and colleagues, they demonstrated that a portion (~33%) of dispersed yeast cells express the hypha-specific hyphal wall protein *HWP1* gene, whereas the yeast wall protein *YWP1* gene was expressed in ~64% of the dispersal yeast cells [23]. It is intriguing that up to a third of the lateral yeast cells express *HWP1* and although the underlying cause is still yet unknown, we postulate that this may be a clever strategy for the fungus so that these *HWP1*-expressing cells are “primed” and ready to colonize and invade the host cells as soon as they land on another site. The same authors also found that *PES1*, which is essential for yeast cell growth, had upregulated expression in dispersal yeast cells compared to biofilm, presumably as an inducer for generating more lateral yeast cells for dispersal [23].

In previous studies, differential expression analysis of the genes involved in various stages of biofilm production in *C. albicans* was mostly assessed via qualitative reverse transcription PCR (RT-PCR) or sometimes quantitative real-time RT-PCR systems. More recent transcriptomic and genomic approaches for studying regulatory networks involved in biofilm formation and the associated antifungal drug mechanisms are discussed in a later section in this review.

## 3. Transcription Regulatory Network of Biofilm Formation

As discussed in the above section, there are still many unanswered questions pertaining to biofilm formation by *C. albicans* in terms of the pathogenesis and the virulence mechanism. In this section, we will further dissect *C. albicans* biofilm formation in the context of the transcriptional regulatory network involved in this phenomenon.

Based on earlier literature, a master transcriptional regulatory network that consists of six major transcription regulators Efg1, Bcr1, Brg1, Ndt80, Tec1 and Rob1 are involved in controlling the normal process of biofilm formation. These regulators were discovered via screening a mutant library and in vivo studies in animal models [24,25]. Efg1 and Tec1 are involved in cell morphology regulation while Ndt80 is involved in biofilm formation. Meanwhile, Brg1 and Rob1 are present only in a species closely linked with *C. albicans* whereas, for Bcr1, its functions are yet to be fully characterized. Nobile et al deduced that transcription regulators form a complex and interconnected network with more than one thousand genes in controlling biofilm formation [25], where most of the target genes were bound by at least two or more transcriptional regulators. The same authors also surmised that the complexity in the biofilm network could be due to a number of factors including environmental influences and formation of cell memory to coordinate the cooperation between cells in order to maintain the dynamism and stability of the biofilm over generations. The authors also postulate that the complexity in the architecture of regulatory network helps to control the gene expression more precisely. On the other hand, evolutionary analysis suggests that transcriptional circuitry for the biofilm network in *C. albicans* has just evolved recently, where broad changes in the cis-regulatory sequence and regulators such as Brg1 and Rob1 are necessary for this modernized biofilm circuit [25].

A few years later, by screening the expanded library for biofilm formation at four different time points (immediately after adherence, at 8, 24 and 48 h), Fox and his coworkers [26] have identified three new transcriptional regulators involved in the biofilm circuit. These new regulators include Rfx2, Gal4 and Flo8, which are imperative and have specific roles in the development of the biofilm over time. The authors suggest that Flo8 is the most critical regulator identified in addition to the six master regulators identified earlier by Nobile [25], as double deletion mutants of Flo8 had severe disruption of biofilm formation at all time-points and the biofilm formed was similar to those formed by strains that resulted from deletions of any one of the previously discovered master regulators. Additionally, Flo8 is speculated to be a biofilm-specific regulator, as the upregulation of its expression was not affected by the form of reference cells (either yeast or filamentous form). Meanwhile, the authors reported that Rfx2 and Gal4 are involved in the biofilms formed at intermediate time points. Additionally, the authors hypothesized that Rfx2 and Gal4 are negative regulators for biofilms, where the enhancement of biofilm formation was observed in both rfx2Δ/Δ and gal4Δ/Δ mutant strains. Furthermore, through chromatin immunoprecipitation studies, the authors reported that *FLO8* is bound by Efg1, Brg1 and Ndt80 while *RFX2* and *GAL4* are bound by Ndt80. These findings further prove that Rfx2, Gal4 and Flo8 are well integrated into the existing *C. albicans* transcriptional biofilm regulatory network [26].

The pathogenesis of a biofilm often begins with adherence and colonization of *C. albicans* on the cell surface. Thus, analyzing the regulatory processes that occur during *C. albicans* adherence could provide a new paradigm to further understand the initial stage of biofilm formation. Finkel et al first reported that adherence of *C. albicans* on a silicone surface is under the control of 29 transcription factors [27]. Amongst these transcription factors, only mutants for Ace2p, Arg81p, Bcr1p, and Snf5p exhibit the anti-adherence properties in vitro. Meanwhile, the deletion of Zcf28p, Zfu2p and Crz2p is only able to disrupt biofilm formation on catheters in animal models [27]. Lee et al adopted a functional genomic analysis approach to screen for a library consisting of 1481 double barcoded doxycycline-repressible conditional gene expression strains which encompass approximately 25% of the *C. albicans* genome [28]. The authors have identified five important adherence regulators namely *ARC18*, *PMT1*, *MNN9*, *SPT7*, and *orf19.831* where transcriptional repression of these genes impaired the adherence of *C. albicans*. Of all, transcriptional repression of *ARC18* results in the strongest adherence defect and cell wall physiology changes in *C. albicans*. Arc18 is one of the putative members of the Arp2/3 complex. The authors demonstrated that perturbation of the Arp2/3 complex reduces biofilm formation, impairs the adherence process, and increases the cell surface hydrophobicity. Additionally, the disruption of the Arp2/3 complex also leads to hyperactivation of small G-protein Rho1-mediated cell wall related stress pathways, where extensive fungal cell wall remodeling is taking place. Taken together, in this study, the authors have identified a novel molecular mechanism between Arp2/3 complex and Rho1 in regulating *C. albicans* adhesion and biofilm formation [28].

**Figure 1:**
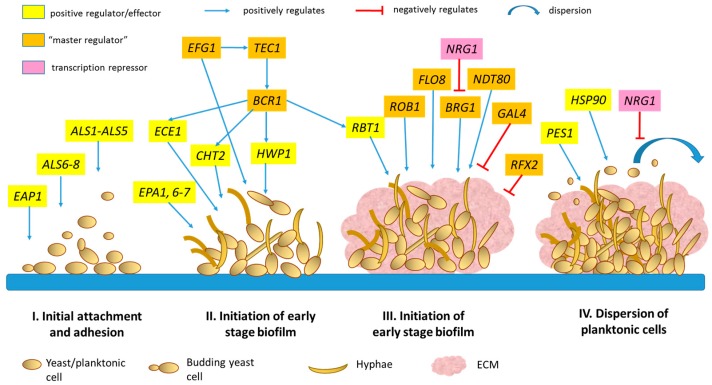
Schematic diagram depicting the stages of biofilm formation in *Candida albicans* and the transcription regulatory network involved in the process. The information on the “master regulators” originated from Nobile [25], Fox [26] and Glazier [29].

Previous findings from Nobile [25], which used genome-wide approaches, have revealed that the transcription regulatory network of biofilm formation is highly integrated. Nevertheless, the functional consequences of this integration and the interactions between transcriptional regulators in this transcriptional circuitry remain obscure. Additionally, the study by Nobile and other studies on transcriptional circuitry for biofilm formation usually employed double homozygous deletion mutants of particular transcriptional regulator genes to study its effect on biofilm formation [24,25,26]. This genetic analysis approach will eventually limit the potential to discover novel genes that contribute towards alterations in biofilm formation, particularly for a single homozygous strain. To address this limitation, Glazier and his workers [29] have adopted a simple haploinsufficiency genetic analysis approach to analyze the transcription regulatory network of biofilm formation. The authors generated and analyzed the interaction between all possible double heterozygous mutants of the transcriptional regulators (Efg1, Bcr1, Brg1, Ndt80, Tec 1 and Rob1). From the study, the authors reported that the biofilm network is remarkably susceptible to genetic perturbation where all of the six transcriptional regulator mutants showed changes in biofilm formation. Additionally, the double heterozygous mutants showed a comparable or more severe disruption in biofilm development than the double homozygous mutants. In this study, the authors also shed light on the functions and involvement of individual transcriptional regulators. The authors revealed that *TEC1* expression is highly sensitive to small disruptions by other transcriptional regulators while *NDT80* expression is under the influence of *TEC1*. Meanwhile, *ROB1* expression was found to be dependent on the cooperative interaction among transcriptional regulators and auto-regulation mechanism [29]. Figure 1 summarizes and highlights the involvement of different transcription factors, master regulators and effectors in the complex biofilm regulatory circuitry according to the distinct stages of biofilm development in *C. albicans*.

## 4. Drug Resistance in *Candida albicans* Biofilm

The five major groups of antifungal agents that are used in the treatment of *C. albicans* infections are azoles, allylamines, echinocandins, polyenes and nucleoside analogues. The azoles and allylamines inhibit the ergosterol synthesis by blocking different enzymes respectively, whereas echinocandins disrupt the cell wall integrity by inhibiting the enzyme β-1,3-glucan synthase. Polyenes bind to sterols and cause intracellular leakage, whereas nucleoside analogues perturb DNA/RNA synthesis thus inhibiting cell growth [30].

Treatment of *Candida* biofilm was shown to be effective with amphotericin B and echinochandins in some previous studies [31,32,33,34,35,36,37]. However, antifungal drug resistance in biofilms of *C. albicans* has been reported in the past few years. Furthermore, the pyrimidine analogs, allylamines and classic formulations of polyenes are not effective against biofilms of *C. albicans* [3,38,39,40]. Factors contributing to antifungal resistance in *C. albicans* have been described. One of the more prominent factors is the ability of this species to effectively pump out drugs via its efflux pumps mechanism [41]. Besides the drug efflux pumps mechanism, the biofilm architecture, which is a thick layer of matrix and its constituents such as the polysaccharide β-1,3-glucan, has the ability to bind to the antifungal agents, preventing them from reaching their targets, which eventually increases the resistance of *C. albicans* towards these drugs [41,42].

Persister cells that exist within the biofilms as metabolically inactive cells are also known to be resistant to many antifungal drugs such as amphotericin B [16,40]. Due to the dormancy of these persister cells, it is difficult for antifungal drugs to evoke an effect as these drugs normally target actively metabolizing cells. In this case, where persister cells exist within the biofilm matrix, much higher minimum inhibitory concentrations (MICs) of the drugs may be required to achieve the intended therapeutic goal. The acquisition of drug resistance in *C. albicans* has been studied using transcriptomes. The RNAseq analyses have been shown to be more accurate and sensitive when compared to microarray and, thus, provide a better platform to unravel the complexity of drug resistance genes in *C. albicans* [43,44]. Using RNA seq, more than 50 genes were found to be overexpressed in drug-resistant *C. albicans* [45]. The transcriptional factor encoded by the *CZF1* gene that is associated with hyphal transition and white/opaque switching was upregulated along with the *CDR1* and *CDR2* genes. This gene is also known to negatively control the expression of one of the three genes which encode β-1,3-glucan synthase, i.e., *GSL1*. Besides these genes, several other genes regulating adherence (*ALS1*), carbon metabolism (*CIT1*, *HGT10*, *GAL7*, *GUT1*, *FUM12* and *GDH3*), cell wall maintenance (*MNN4* and *CHR11*), drug transport (*YOR1*) and morphogenesis (*WHI1*, *ADAEC*, *SFL2*, *SCH9*, *CZF1*, *ECE1*, *DLD1*, *GPR1* and *SRR1*) of *C. albicans* were overexpressed. The study also revealed a few other genes that were repressed such as gene regulating adherence (*ALS2*), carbon metabolism (*HGT12*, *MAL2* and *MAE1*), copper and iron homeostasis (*HEM13*, *CRP1* and *SMF3*), drug transport (*CDR4* and *MFS*), extracellular proteins (*PLB1*, *ALS2* and *MAL2*), morphogenesis (*NAT4* and *PHHB*) and steroid binding (*EBP1* and *CBP1*). A new transcribed region was identified upstream of the *TAC1* gene, which encodes the major *CDR* transcriptional regulator, which is yet to be characterized [45].

## 5. Comparative Studies on *C. albicans* vs. “Avirulent” *C. dubliniensis*

*C. albicans* is the most commonly found opportunistic yeast that can exist as a normal microflora in healthy individuals or as an etiological agent in human candidiasis. *C. dubliniensis*, first identified in 1995, is a pathogenic species that is phylogenetically close to *C. albicans* [46,47]. Similar to *C. albicans*, *C. dubliniensis* is able to form a chlamydospore and germ tube and to cause oral candidiasis, particularly in human immunodeficiency virus (HIV)-infected patients [48]. Despite the phylogenetic similarities between *C. dubliniensis* and *C. albicans*, the former exhibits a poorer virulence and lower prevalence rate due to the reduced capability to colonize the host and to form filaments [48,49,50]. Previous studies have focused on elucidating the phenotypical differences between these two closely related species to understand the fungal virulence mechanism. Some of the prominent findings from these studies indicate that *C. dubliniensis* exhibits impaired growth kinetics at 42 °C [51], has no β-glucosidase activity [52] and fails to produce true hyphae under *N*-acetylglucosamine stimulation or a nutrient-rich environment [46,53]. However, it shows a higher extracellular proteinase expression, adheres more strongly to buccal epithelial cells and is less susceptible to 5-flucytosine compared to *C. albicans* [54].

Using an in vivo animal infection model, Vilela demonstrated that mice intravenously infected with *C. dubliniensis* show higher survival rates than those infected with *C. albicans* and this is likely due to a more effective host inflammatory immune response to *C. dubliniensis* [48]. This is supported by in vitro culture experiment whereby *C. dubliniensis* had a lower survival rate in the presence of human polymorphonuclear leukocytes [55]. Furthermore, in an immunosuppressed mouse model, Stoke demonstrated that *C. dubliniensis* poses a weaker degree of expansion and dissemination to internal organs than *C. albicans* [50]. When co-cultured with oral reconstituted human epithelial cells, *C. dubliniensis* appears predominantly in yeast form that renders minimal effect to the cells; whereas *C. albicans* forms abundant hyphae that damage the epithelial cells in vitro [50].

**Figure 2:**
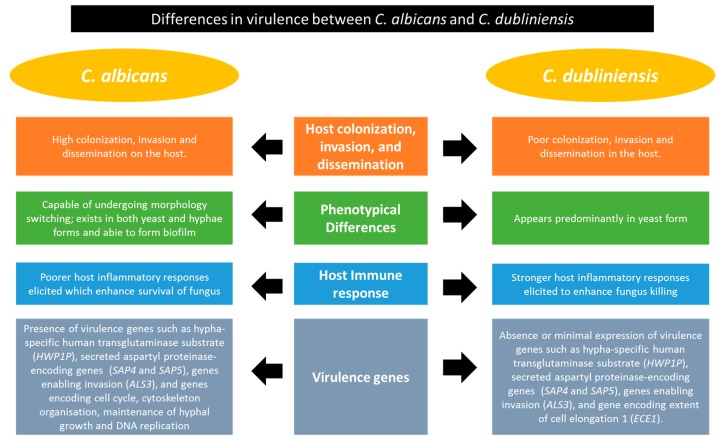
Comparative differences in the virulence determinants between *C. albicans* and *C. dubliniensis*. Differences in (i) host colonization, invasion, and dissemination; (ii) morphology; (iii) host immune response; and (iv) virulence genes are highlighted.

Given that *C. albicans* and *C. dubliniensis* share a close phylogenetic relationship, the puzzle remains as to how these two species are varied in terms of virulence. Studies have used genomics and transcriptomics approaches to gain further insights into the different morphogenesis pathways in both species [53,55,56,57]. Moran has a utilized comparative genomic hybridization (CGH) method by co-hybridizing *C. albicans* microarrays with fluorescently labelled *C. albicans* and *C. dubliniensis* genomic DNA to assess the cross species genomic homology [55]. The outcome from this experiment shows a high genome similarity between the two species. Up to 95.6% of *C. dubliniensis* genomic DNA is homologous to, and hybridizes with, nucleotides from the *C. albicans* genome; while only a small proportion (4.4%, 247 genes) shows sequence divergence. The divergent genes include those encoding the hypha-specific human transglutaminase substrate *HWP1P*, which are important for hyphae formation. It is also noted that two of the *C. albicans* virulence factors implicated in invasion, the secreted aspartyl proteinase-encoding genes (*SAP5*, and either one of the *SAP4* or *SAP6*), are missing in the *C. dubliniensis* genome. Further, Jackson and coworkers sequenced the 14.6-megabase genome of *C. dubliniensis* and compared it to that of *C. albicans* using whole-genome shotgun sequencing at 11-fold average coverage [56]. Similar to results from Moran [55], that utilize the CGH method, a highly conserved sequence and synteny are shown throughout the genome of both species and only a total of 168 species-specific genes are identified. The absence of *SAP4* and *SAP5* genes in *C. dubliniensis* genome is reconfirmed in this study. Other genes reported in this study include the proposed invasin *ALS3* and a group of 115 pseudogenes that are orthologs of filamentous growth regulator (*FGR*) genes with predicted functions in fungal pathogenesis. A study by Butler compared eight *Candida* species (without *C. dubliniensis*) and shows some genes involved in mating and meiosis pathways are missing throughout evolution in certain species [58]. This suggests that distinct *Candida* species may have modified their genomes during evolutionary adaptation, which may contribute to the different virulence levels of each species. Figure 2 provides a summary of the differences between *C. albicans* and *C. dubliniensis* in different aspects, which contribute to the differences in virulence.

Compared to the genomic data, transcriptomic analysis provides more vital information of transactive genes under different stimuli or conditions. Transcriptomic analysis has been used to analyze the cross species gene expression between *Candida* versus other fungal species [57,59,60,61]. In 2013, Grumaz [57] used RNA-sequencing (RNA-Seq) on the Illumina (San Diego, CA, USA) next generation sequencing platform to compare the transcriptional landscapes between *C. dubliniensis* and *C. albicans* in both hyphal and yeast stages. A comparison of the differentially expressed orthologs was quantitatively determined. Recently, an RNA-Seq approach has also been used to compare the transcriptome of *C. albicans* versus *C. africana*, a biovariant with a low degree of virulence and inability to produce chlamydospores; which unveiled two novel transcriptionally active regions in both species [61]. A recent study by Caplice [53] uses a microarray meta-analysis to compare the transcriptional response of *C. dubliniensis* and *C. albicans* to different stimuli such as pH and temperature [53]. Interestingly, *C. dubliniensis* displays no or minimal expression of several Efg1-regulated, hypha-induced genes, such as the extent of cell elongation 1 (*ECE1*) and *HWP1* in response to 37 °C incubation. Other genes that are induced in *C. albicans* but not *C. dubliniensis* include those involved in the cell cycle, cytoskeleton organization, and the maintenance of hyphal growth and DNA replication. Hence, -omics approaches to compare between a less virulent and less versatile pathogen *C. dubliniensis* (or *C. africana*) to the highly virulent *C. albicans* provide important clues for the intricate gene regulatory network of the virulence process.

## 6. Exploitation of Transcriptomic and Genomic Technologies for Dissecting *C. albicans* Biofilm Formation and Drug Resistance

The *C. albicans* genome was sequenced and annotated via the efforts of the Stanford Genome Technology Centre as well as the European Galar Fungail network. The CandidaDB genome database for *C. albicans* pathogenomics was first launched in January 2002. Later, the release of CandidaDB launched in June 2004 represented an up-to-date annotation of Assembly 19 of the *C.albicans* genome sequence [62]. The genome database provided a strong impetus for and an indispensable base, which spurred numerous whole-genome related transcriptomics and proteomic studies. Table 1 below is a summary of the key publications within the last decade, which helped to advance our knowledge in *C. albicans* biofilm formation and drug resistance, particularly through those that describe the use of new -omics platforms. The list is in no way exhaustive but merely a snapshot of the studies that have contributed to this field.

## 7. Future Directions of Research on New Antifungal Drugs Targeted at *Candida* Biofilm

Undoubtedly, biofilm formation by *C. albicans* is a complicated process leading to life-threatening infections, which are difficult to eradicate. Additionally, current antifungal therapies have minimal effects on biofilm formation and there is no effective solution to solve this problem. Moreover, the development of resistance and toxicity further hindered the efficacy of antifungal drugs. Nonetheless, there are some promising strategies that could be carried out to tackle biofilm formation. These strategies include lock therapies for an infected catheter, catheter coatings, natural products and synthetic products screening, photodynamic inactivation, and targeting of the molecular pathways related to *C. albicans* biofilm formation.

The “lock therapy” is where high concentrations of antifungal drugs are directly administered into the catheter lumen for a period of time, such as several hours to days, prior to contact with patients. This approach allows the antifungal agent to eliminate biofilm formation in the catheter and also to avoid undesirable systemic toxicity build-up in patients as the high dosage of the antifungal agent only acts in the catheter [67]. This therapy has been tested on silicone catheters infected with different *C. glabrata* and *C. albicans* strains, using caspofungin, micafungin and posaconazole as antifungal agents. The outcomes were positive where all antifungal agents used in the study have successfully reduced biofilm formation, with micafungin showing the most promising result [68]. Meanwhile, another lock therapy using amphotericin B lipid formulation (L-AMB) was not effective to eradicate *Candida* biofilms [69]. There are several concerns when considering lock therapy in catheter-related candidemia including (1) the higher failure rate for biofilm infection on the outer surface or on the catheter tip; (2) the possibility of the development of antifungal drug resistance and (3) concomitant systemic antifungal treatment may need to be considered for disseminated infection. To counter these concerns, the choice of non-antifungal agents or antiseptics such as ethanol, EDTA or a high dose of minocycline are more preferable in the lock therapy against *C. albicans* and non-*albicans* biofilms [70].

Another alternative to counter *C. albicans* biofilm formation is through the modification of the catheter coating. A study has shown that the modification of the catheter coating with chlorhexidine, minocycline-rifampin or silver sulfadiazine could reduce the incidence of bloodstream infections caused by central venous catheters in the intensive care unit [71]. Meanwhile, a novel silane system coated on the implant surfaces of titanium and zirconia has been shown to inhibit *C. albicans* biofilm formation [72]. On the other hand, Karlsson fabricated and coated antifungal β-peptide-containing multilayered polymer films onto surfaces and demonstrated a profound inhibition of the antifungal-containing polymer films on the growth and proliferation of *C. albicans*. The authors suggest that this approach could be used to suppress the biofilm formation caused by *C. albicans* on film-coated surfaces, which could be further applied onto the surface of medical devices to inhibit *C. albicans* biofilm in clinical settings [73]. Hoque also demonstrated that by coating the surface of medical devices with water-insoluble and organo-soluble polymeric materials inhibition on the growth and proliferation for a number of bacteria and fungi including *C. albicans* was remarkable [74].

Exploiting the efficacy and synergistic effect of combinations of antifungal therapies with other drug classes could be another strategy for new antifungal development against *C. albicans* biofilm formation. For example, synergistic effects between cyclosporine A with fluconazole, caspofungin, voriconazole, nystatin and amphotericin B against *C. albicans* biofilm formation were observed [75]. Additionally, our previous work also demonstrated that allicin, a pure compound from garlic extract, when combined with fluconazole reduced the *C. albicans* biofilm formation and altered the expression of biofilm-related genes in vitro [76]. Besides that, the combination of Hsp90 inhibitors and the non-steroidal anti-inflammatory drugs (NSAIDs) with antifungal drugs also showed a promising synergistic activity against *C. albicans* biofilm formation [77,78]. The additive effects of different drugs such as that between fluconazole and doxycycline were also effective against *C. albicans* biofilms [79]. Nevertheless, the possibility of the development of cell toxicity has to be considered when adopting such an approach.

On the other hand, formulation of new drugs based on existing antifungal drugs could be another approach against *C. albicans* biofilm formation. Hitherto, several new formulated drugs, including an amphotericin B lipid complex and liposomal amphotericin B, showed efficacy against *C. albicans* biofilm in a bioprosthetic model [32]. Caspofungin, echinocandin, and micafungin also showed similar effects against *C. parapsilosis* and *C. albicans* biofilms in the same model [32]. Recent studies also documented the efficacy of amphotericin B lipid and echinocandin against *Candida* biofilm formation both in vitro (34) and in vivo (35,37). Meanwhile, aspirin was shown to inhibit *C. albicans* filamentation and biofilm formed by *C. albicans*, *C. parapsilosis* and *C. glabrata* [80]. Taken together, future work on the discovery of new antifungal drugs through in silico modeling or structural modification of existing antifungal drugs can help us in reducing the emergence of antifungal drug resistance.

Targeting the biofilm-related pathways in *C. albicans* could serve as a promising strategy in combating *C. albicans* biofilm formation. One instance is targeting the quorum-sensing pathway. Farnesol is a quorum-sensing molecule involved in facilitating the communication between *Candida* cells during cell proliferation. Previous studies have shown that farnesol is able to inhibit *C. albicans* biofilm formation and augment the efficacy of azoles [22,81,82,83]. Further evaluation of the efficacy of farnesol in vivo and the discovery of more quorum sensing molecules could shed light on the possibility of targeting the quorum-sensing pathway for *C. albicans* biofilm treatment. Meanwhile, targeting the biofilm ECM of *C. albicans*, such as β-1,3 glucan and extracellular DNA (eDNA) could be another potential approach for anti-biofilm therapy. As such, studies have shown fluconazole activity is enhanced upon digestion of β-1,3 glucan while amphotericin B activity is enhanced by the degradation of eDNA [11,42,84]. More studies identifying potential inhibitors for targeting the biofilm matrix and pathways could help us in designing better antifungal drugs for anti-biofilm therapy.

On-going and intense research has explored natural products or synthetic peptides against *C. albicans* biofilm formation. Compounds under investigation include terpenoids, polyphenols and phenylpropanoids from plant and tea extracts [85,86] and also phenazines produced by *P. aeruginosa* [87]. These compounds can suppress *C. albicans* biofilm formation and inhibit yeast-to-hyphal transition. Additionally, some synthetic peptides, such as KSL-W have significant effects on biofilm formation, growth and yeast-to-hypha transition of *C. albicans* [88]. On the other hand, several high-throughput screenings aimed at identifying small-molecule inhibitors against *C. albicans* filamentation and biofilm formation have been conducted. Siles screened for 1200 off-patent drugs approved by the Food and Drug Administration within the Prestwick Chemical Library and identified 38 bioactive compounds with ability in suppressing *C. albicans* biofilm [89]. Subsequently, Wong screened for 50,240 small molecules from a library and identified SM21 as a potent inhibitor for *C. albicans* yeast-to-hypha transition [90]. Pierce identified a series of diazaspiro-decane structural analogs which inhibit the filamentation and biofilm formation of *C. albicans* from a chemical library (NOVACore™) of 20,000 small molecules [91]. Meanwhile, Romo screened for 30,000 small-molecules within ChemBridge’s DIVERSet chemical library and identified *N*-[3-(allyloxy)-phenyl]-4-methoxybenzamide as the leading compound for preventing *C. albicans* filamentation and biofilm formation [92].

Another potential approach to eliminate *C. albicans* biofilm is through photodynamic inactivation. This technique adopts the use of a nontoxic dye (photosensitizer) and visible light to produce reactive oxygen species, which are able to destroy the DNA, cell membrane or proteins of microbial cells and subsequently kill them. Several photosensitizers have been tested on *Candida* biofilm formation including methylene blue and toluidine blue [93,94]. A study has shown that toluidine blue (0.1 mg/mL) is able to reduce *C. albicans* biofilm at up to 60% [93]. In addition to that, when combined with chitosan, a greater reducing effect on *C albicans* biofilm formation was observed [95]. This non-toxic and yet cost-effective technique will definitely serve as a future expansion field in reducing biofilm formation of *Candida* spp.

In recent years, nanoparticles with antifungal properties have been described. There is rife interest on the anti-biofilm properties of silver (Ag) nanoparticles, which have been shown to damage the cell wall and membrane of *C. albicans* biofilm cells and inhibit filamentation [96]. A recent study also showed that Ag nanoparticles have altered multiple cellular targets including ergosterol content, fatty acid composition, cell membrane integrity and ultrastructure [97]. Another biopolymer with promising anti-biofilm property is chitosan nanoparticles, which were demonstrated by a recent study to be effective in *C. albicans* biofilm inhibition on a resin denture surface [98]. Moreover, the efficacy of other nanoparticles namely gold nanoparticles [99], silica nanoparticles [100] and selenium nanoparticles either alone or as a carrier for antifungal drugs has also been explored [101].

Vaccination is another potential strategy in preventing invasive fungal infections, particularly on high-risk groups with identifiable risk factors [102,103]. The key protection lies in the ability of vaccines to boost host immunity, including pro-inflammatory, cell-mediated, Th1 or Th17 responses to enhance phagocytic killing of the fungus [103]. Dedicated studies have been conducted by researchers in recent years to develop safe and effective fungal vaccines [104,105]. Though no specific vaccine has been developed to prevent *Candida* biofilms, however, two promising fungal vaccines against invasive candidiasis have been developed and they are currently under clinical trials. The first *Candida* vaccine containing the rAls3p-N antigen is presently under a phase IIa clinical trial whereby the results indicated that this vaccine hinders fungal adhesion and invasion in immunized subjects [105,106]. The second *Candida* vaccine is a virosome-based vaccine comprising of Sap2 antigen/truncated recombinant Sap2 antigen, which confers protection for both systemic and mucosal candidiasis [107]. Concerted efforts should be given to unravel new compounds and molecules that can be applied in the prophylaxis and treatment of *Candida* infections.

In conclusion, the majority of the data generated on *C. albicans* biofilms has mainly relied on in vitro models, which pose limitations in translating the findings from bench to bedside. Each in vitro biofilm model could be limited by the species/strains used, the specific environmental conditions and the choices of biotic interphase. It is important to take into consideration the clinical relevance of the adopted model as well. The lack of in vivo studies also warrants the development of reliable and novel in vitro biofilm models that resemble the conditions in vivo, which can be utilized for long-term anti-biofilm and antimicrobial activity prediction. On the other hand, from the accumulated data derived from transcriptome expression profiles of *C. albicans* biofilm, various key regulators of biofilm development have been identified. Dispersal cells, in particular, should be targeted as they are programmed to survive in nutrient-starved niches and to infect new sites in the host. Using the latest molecular docking and in silico modeling software to screen libraries of small molecules and peptides for candidates that could bind to and inactivate selected CSTARs targets and key regulators such as *PES1, YWP1*, *HWP1* and the Arp2/3 complex, we might be able to identify potential anti-*C. albicans* molecules that could become the next marketed antifungal drugs.

## Figures and Tables

**Table 1:** Gene expression studies on *C. albicans* biofilm and drug resistance.

Methodology/Platform	Biological Issue Studied	Major Findings	Reference
Microarrays with probes for *C. albicans* genes/Eurogentec (Seraing, Belgium)	Transcription profiles of biofilm cells vs. planktonic cells under different conditions of flow, oxygenation, and glucose concentration	Gcn4p, a regulator of amino acid metabolism, is required for biofilm growth	[63]
Microarrays/Eurogentec SA (Ivoz-Ramet, Belgium) in collaboration with European Galar Fungail Consortium (www.pasteur.fr)	Genome-wide expression profile of *C. albicans* to polyene, pyrimidine, azole, and echinocandin antifungal agents	Different expression profile signatures obtained in exposure to different classes of antifungals with various genes overexpressed	[64]
Oligonucleotide microarray (Agilent Technologies, Santa Clara, CA, USA) with *C. albicans* Assembly 21 genome (http://www.candidagenome.org/), Full Genome Chromatin Immunoprecipitation Tiling Microarray (ChIP-chip), RNA Sequencing (RNA-seq)	Comparative transcriptional analysis of *C. albicans* biofilm and planktonic cells, with *C. albicans* transcription regulator (TR) deletion mutants that are deficient in biofilm formation	Six master regulators Bcr1, Tec1, Efg1, Ndt80, Rob1, and Brg1 are essential for biofilm formation.	[25]
NanoString expression profiling and nCounter platform (NanoString Technologies, Inc., Seattle, US) with ~150 probes from cell wall-related genes, ~50 host-pathogen interaction genes, ~100 genes highly regulated during hypha development or biofilm formation, oxidative or osmotic stress	Expression profiling of genes involved in *C. albicans* adherence to substrate (silicone), an early step in medical device-related infections	Biofilm regulators Bcr1 and Ace2 have a role in adherence. A large regulatory network of 11 adherence regulators, the zinc-response regulator Zap1, and approximately 25% of the predicted cell surface protein genes known as Cell Surface Targets of Adherence Regulators (CSTARs) are involved in adherence.	[27]
RNA-sequencing (mRNA-Seq 8, Illumina) and Genome Analyzer (Illumina Inc.)	Transcriptome analysis of a *Candida* Drug Resistance (*CDR*) strain against its isogenic drug susceptible counterpart	Identified ∼50 genes overexpressed in *CDR* strain: *CZF1* which is involved in transcription regulation of white/opaque switching and hypha formation is upregulated with *CDR1* and *CDR2*, 5’UTR region of TAC1	[45]
Gene expression microarrays (Agilent Technologies), Chromatin immunoprecipitation quantitative real-time PCR (ChIP-qPCR)	Genome-wide expression analysis of biofilm formation at different intervals, immediately after adherence, at 8, 24 and 48 h	Identified Flo8, Gal4, and Rfx2 to be involved in different time points of biofilm formation	[26]
High-throughput next-generation sequencing/Hi-Seq 2500 platform (Illumina)	Use pooled Gene Replacement and Conditional Expression (GRACE) library conditional expression strains to identify novel regulators of cell-to-surface adherence	Novel functional relationship between the Arp2/3 complex and Rho1 important for modulating actin cytoskeleton, endocytosis and cell wall remodeling,	[28]
RNA-seq with TruSeq RNA v2 kit/HiSeq2500 platform (Illumina)	Transcriptomic profiling of 124 mutant *C. albicans* strains in 10 in vitro conditions for filamentation ability	Genes encoding cell wall/membrane proteins, adhesins, alcohol dehydrogenases, and iron uptake and utilization genes were common genes upregulated across different conditions	[65]
*Candida* genome microarray (CapitalBio Corp., Beijing, China) with *C. albicans* genome database (http://www.candidagenome.org/)/CapitalBio BioMixer II hybridization station	Gene expression profiling of fluconazole-resistant *C. albicans* strain treated with osthole (a natural coumarin) in synergy with fluconazole	Genes in oxidation-reduction process (e.g., catalase encoded by *CAT1* and mitochondrial glycosylase encoded by *OGG1*); *CTN1* (carnitine acetyltransferase) and *ICL1* (isocitrate lyase) were upregulated	[66]
RNA-seq with BIOO Scientific NEXTflex Directional RNA-seq kit/HiSeq2000 platform (Illumina)	To decipher transcriptional gene expression patterns of dispersal cells versus core biofilm cells and planktonic cells	Transcription pattern of dispersal cells mostly similar to parent biofilm, *YWP1* expression ~2-fold higher in dispersal > biofilm > planktonic cells; ~33% of dispersal yeast cells express *HWP1*	[23]
